# The Anode Challenge for Lithium‐Ion Batteries: A Mechanochemically Synthesized Sn–Fe–C Composite Anode Surpasses Graphitic Carbon

**DOI:** 10.1002/advs.201500229

**Published:** 2016-02-04

**Authors:** Zhixin Dong, Ruibo Zhang, Dongsheng Ji, Natasha A. Chernova, Khim Karki, Shawn Sallis, Louis Piper, M. Stanley Whittingham

**Affiliations:** ^1^ Materials Science and EngineeringState University of New York at BinghamtonBinghamtonNY13902‐6000USA; ^2^ Department of ChemistryState University of New York at BinghamtonBinghamtonNY13902‐6000USA; ^3^ Center for Functional NanomaterialsBrookhaven National LaboratoryUptonNY11973USA

**Keywords:** anode, high energy ball mill, lithium‐ion battery, Sn_2_Fe, volumetric capacity

## Abstract

Carbon‐based anodes are the key limiting factor in increasing the volumetric capacity of lithium‐ion batteries. Tin‐based composites are one alternative approach. Nanosized Sn–Fe–C anode materials are mechanochemically synthesized by reducing SnO with Ti in the presence of carbon. The optimum synthesis conditions are found to be 1:0.25:10 for initial ratio of SnO, Ti, and graphite with a total grinding time of 8 h. This optimized composite shows excellent extended cycling at the C/10 rate, delivering a first charge capacity as high as 740 mAh g^−1^ and 60% of which still remained after 170 cycles. The calculated volumetric capacity significantly exceeds that of carbon. It also exhibits excellent rate capability, delivering volumetric capacity higher than 1.6 Ah cc^−1^ over 140 cycles at the 1 C rate.

## 1 Introduction

Lithium‐ion batteries, which have become one of the most important energy storage sources, have been successfully applied in a wide range of applications, including portable electronic devices, medical devices, electric vehicles (EV), hybrid electric vehicles (HEV), and grid storage.^1^ Graphitic carbon has been widely used as the anode material in commercial lithium‐ion batteries, due to its low cost, stable cycling, and long cycle life. However, carbon has a limited volumetric capacity of 740 Ah L^−1^ because of its low gravimetric density, making the carbon anode the major impediment in increasing the volumetric capacity of lithium‐ion batteries.^2^ Moreover, high rate charging is hazardous for carbon anodes since lithium dendrite formation might occur and cause cell short circuit.^2^ Therefore, better anode materials are needed to meet the demands of future lithium‐ion battery applications. Tin‐based materials are believed to be a potential substitute for carbonaceous anodes due to their higher packing density, higher gravimetric/volumetric capacity, and safer thermodynamic potential compared to the carbonaceous anode materials.^3^ As a result, much research effort has been invested in tin‐based anodes. However, the inevitable large volume change (>300%) of tin‐based anodes during the lithiation/delithiation process leads to particle cracking and subsequent separation of the active materials from the current collector. This results in a rapid capacity fade, and thus hinders the practical application of such materials in real‐world batteries.^4^ To mitigate the volume expansion, various strategies have been applied, including nanostructuring, building composites, and so on.^5^ Nanosized material can partly solve the problem of pulverization, shortening the lithium diffusion path^6^ and slowing down the capacity fade;^7^ however, the accompanying high surface reactivity, low tap density, as well as the flammable or explosive tendency are challenges that need addressing.^8^ Building composites by dispersing tin in an active or inactive matrix (Sn‐M) is another strategy to improve the behavior of tin‐based materials, which could help buffer the large volume expansion and thus enhance the cyclability.^9^ Hence, significant efforts have been placed on the Sn‐M (M = Co, Cu, Ti, Fe, etc.) composite system^10^ and its electrochemical behavior is improved over pure tin. In addition, carbon is often added in this Sn‐M system since it can benefit the cycle life.^11^

In 2005, a new lithium‐ion battery with the anode consisting of amorphous Sn‐Co‐C was launched by Sony Corporation under the commercial name of Nexelion.^12^ It delivered a reversible capacity of more than 500 mAh g^−1^ at 1 mA cm^−2^ over 30 cycles.^13^ The Sn‐Co‐C composite material was also prepared through a simple solution polymerization method followed by pyrolysis by Li et al.^14^ the capacity of which attained 700 mAh g^−1^ over 100 cycles under a current density of 50 mA g^−1^. Amorphous Sn‐Cu‐C was investigated by Thorne et al. and a stable cycle life of 100 cycles at 400 mAh g^−1^ was obtained.^15^ Titanium was also used as matrix to form Sn‐Ti‐C composite, and a stable capacity of 370 mAh g^−1^ was achieved over 300 cycles^16^ by Yoon and Manthiram. Sn‐Fe‐C composites with different composition were prepared according to Gibbs composition triangle[[qv: 4a,17]] in Dahn's group. Sn_2_Fe had the highest reversible capacity among the binary Sn‐Fe phases they tested.[[qv: 17a]] When it comes to the Sn_2_Fe‐C system, a reasonable cycle life and a reversible capacity of ≈200 mAh g^−1^ was achieved when the voltage window was restricted to 0–0.55 V.[[qv: 4a]] An Sn_2_Fe‐SnFe_3_C‐C composite was also investigated carefully by Dahn's group and they claimed the best material they got had a volumetric capacity of 1600 mAh cm^−3^ over 80 cycles, however, the gravimetric capacity was low (≈200 mAh g^−1^).[[qv: 17b]]

As discussed above, among all the Sn‐M‐C composites, the Sn‐Fe‐C material is the most promising for large‐scale production since iron is low cost, environmental benign, and earth‐abundant, especially compared to the expensive and toxic cobalt used in Sony Nexelion. Besides, comparing to other stable binary Sn‐Fe alloys, Sn_2_Fe has tunnels around the Sn atoms which allows lithium atoms to reach Sn and initiate the alloying process.[[qv: 17a]] Also, the iron content in Sn‐Fe alloy should be kept low because more iron content might cause an impenetrable “skin” of Fe atoms during the electrochemical cycling which may prevent a full reaction of Sn with Li, resulting in a lower capacity than expected.[[qv: 17a]] Although the Sn_5_Fe composition was reported by Wang et al.^18^ recently, the yield is very low and to the best of our knowledge, no other facile ways of making this phase have been reported. As a result, Sn_2_Fe is the most feasible choice to be utilized as lithium‐ion battery anodes among all the Sn‐Fe phases.

It had been previously reported by our group that the Sn‐Fe‐C composite could be prepared by a mechanochemical method, which is low‐cost, facile, and easily scaled up.^19^ The problem of poor capacity retention brought up by iron‐containing intermetallics^20^ was ameliorated by the addition of graphite. However, the key factors which affect the electrochemical behavior of the final product, such as the reaction time, source of carbon, ratios of initial reactant, and so on have not been determined. To understand how these synthetic factors influence the properties of mechanochemically synthesized Sn‐Fe‐C anode, we hereby carried out a systematic optimization on several synthesis conditions. Finally, the critical factors were determined and we show here that the optimized Sn‐Fe‐C composite is a very promising anode for the next generation lithium‐ion batteries.

## 2 Results and Discussion

### 2.1 Total Grinding Time

**Figure 1:**
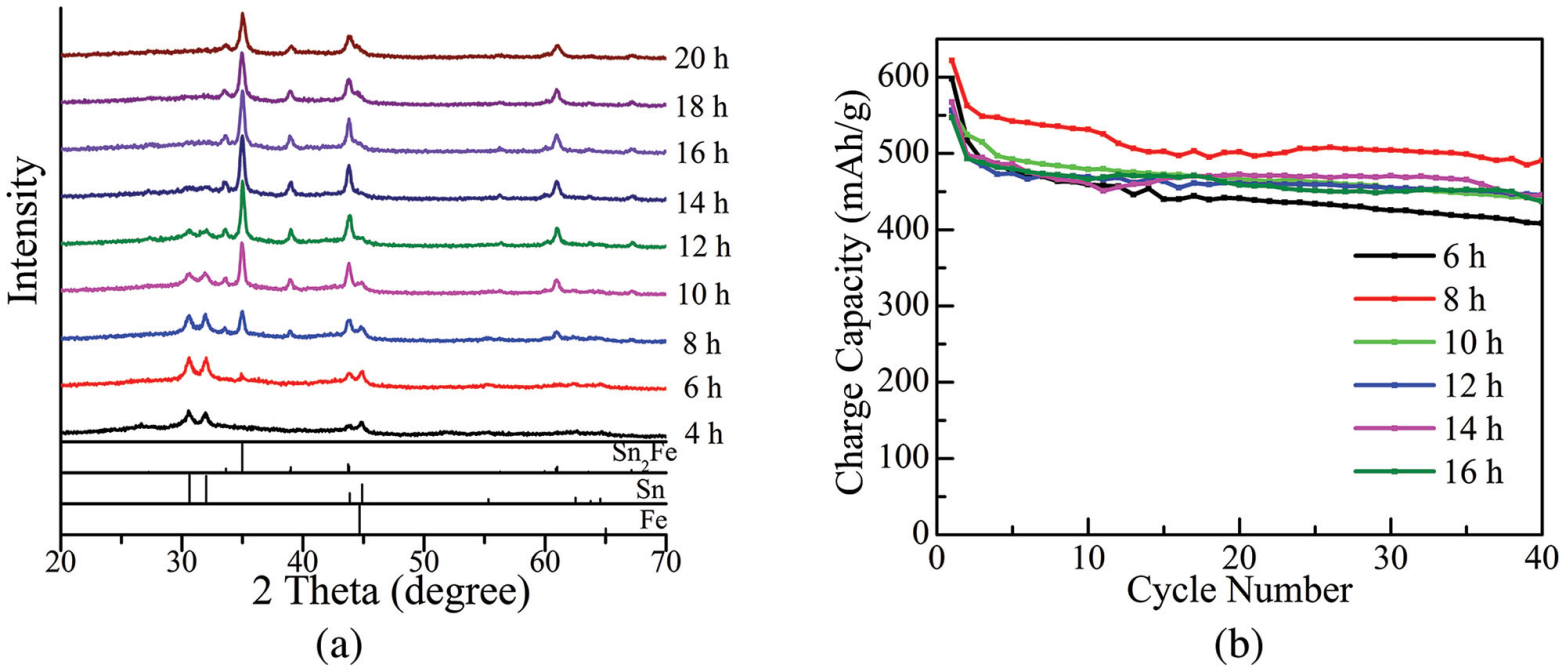
a) XRD patterns and b) cycling performances (cycled under C/10) of nanosized Sn‐Fe‐C anode materials reduced by titanium with different ball milling time and hard balls as grinding media.

It was found in our previous study that using titanium as the reducing agent yielded good electrochemical performance for this ball‐milled Sn‐Fe anode material.^19^ However, the key factors of this synthetic approach and how they govern the electrochemistry were not elucidated. For this reason, X‐ray diffraction (XRD) was performed on the samples reduced by titanium with different ball milling time from 4 to 20 h, and hard balls were used as the grinding media. The results are shown in **Figure** **1**a.

It can be seen in Figure 1a that crystalline Sn_2_Fe phase is clearly formed after 6 h, while tin is found when the reaction time is less than 14 h. The diffraction peaks of Sn disappear gradually while the peaks of Sn_2_Fe become sharper as the reaction time increases from 6 to 16 h, which indicates that the tin generated from SnO reduction converted to Sn_2_Fe as the reaction time increases. Steady state is reached after ≈14 h of ball milling, by which time barely any crystalline tin can be identified, yet the iron phase still keeps forming. Since no observable Sn_2_Fe phase formed for reaction times less than 6 h and no change was seen in the XRD patterns from the electrochemical active phases (Sn and Sn_2_Fe) after 16 h, cycling tests were performed on the samples synthesized between 6 and 16 h, which is shown in Figure 1b.

From the electrochemical performance point of view, the 6 h sample, which has a significant amount of tin shows the worst capacity retention. The 8 h material had the best capacity retention, and increased grinding time resulted in lower capacity retention. This material showed the presence of both Sn_2_Fe and Sn, which indicates that essentially pure Sn_2_Fe (14+ h grinding) is not advantageous. Based on the overall electrochemical behavior, the optimum reaction time is found to be 8 h. Capacity retention is very good after the first few cycles.

Scanning electron microscopy (SEM) and transmission electron microscopy (TEM) images show that the primary particles of less than 100 nm aggregate to form micrometer‐sized secondary particles. Longer reaction times lead to larger aggregates of smaller nanoparticles (see Figure S1, Supporting Information). Analysis of the XRD results using the Scherrer equation,^21^ are consistent with crystallite sizes of around 100 nm. These nanosized particles favor electron transfer as well as lithium diffusion as compared to big particles due to the shorter diffusion distances.^6^ The morphology of the products synthesized with different reaction time can be found in Figure S1 (Supporting Information).

Considering the combination of these XRD, SEM, TEM, and electrochemical tests, the optimum reaction time of 8 h was chosen for the study of the other critical parameters, unless otherwise indicated.

### 2.2 Grinding Media

The grinding media also plays an important role in this mechanochemical reaction. Therefore, the same synthesis procedure as used for the hard balls was performed with soft balls as grinding media, and XRD was performed on the resulting products prepared with different time to better understand the impacts of different grinding media.

**Figure 2:**
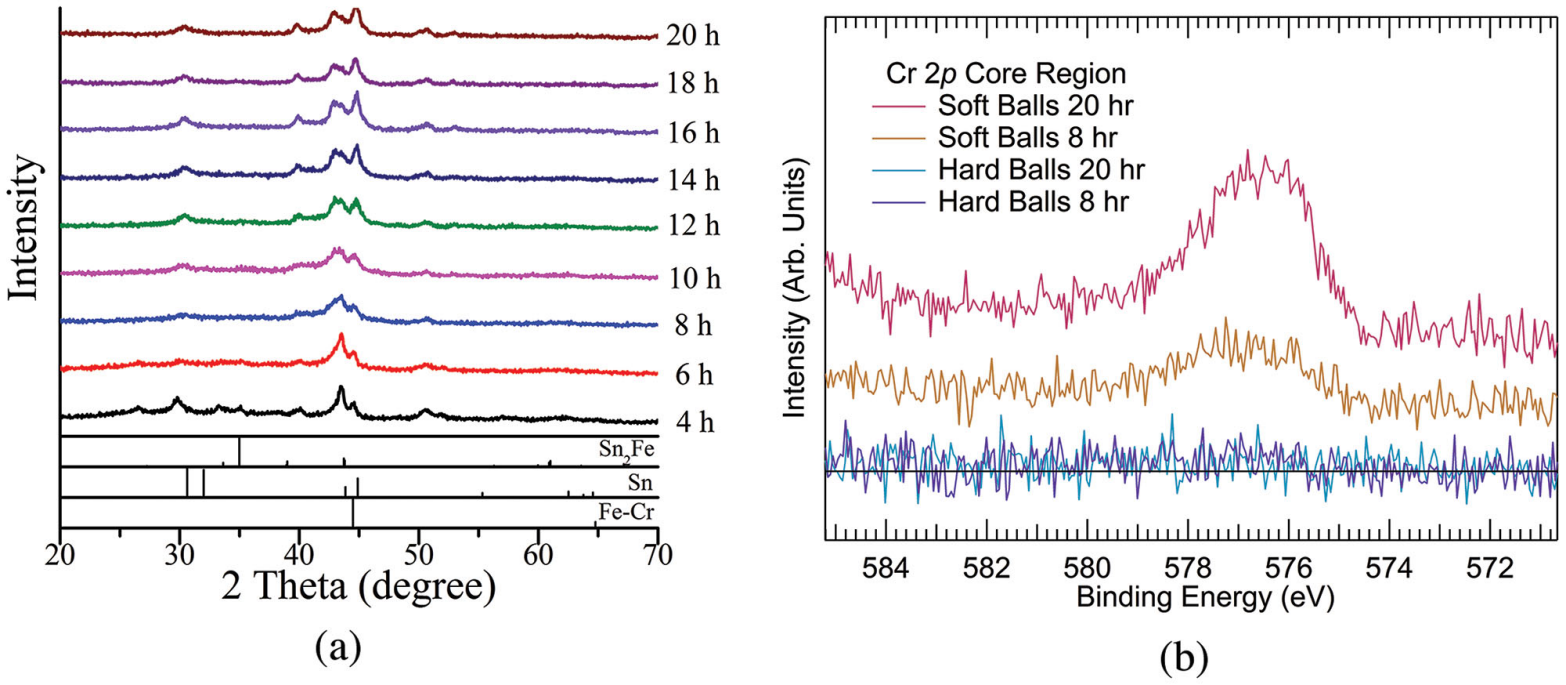
a) XRD patterns of nanosized Sn‐Fe‐C anode materials reduced by titanium with different ball milling time and soft balls as grinding media, compared with the JCPDS files, the total ball milling time is indicated next to each curve and b) Cr 2p core region XPS spectra of selected Sn‐Fe‐C samples.

As shown in **Figure** **2**a, when SnO is reduced by Ti by grinding with soft iron balls, the strong Sn_2_Fe peak at around 35° 2*θ* is absent, which differs from our previous results.^19^ This difference could be attributed to the different type and quantities of soft balls used. The additional peaks around 44°–45° 2*θ* can be attributed to an Fe‐Cr phase coming from the soft balls. The presence of Cr was confirmed by X‐ray photoemission spectroscopy (XPS) spectra (Figure 2b); no Cr‐related phase was found in the material formed with the hard balls.

**Figure 3:**
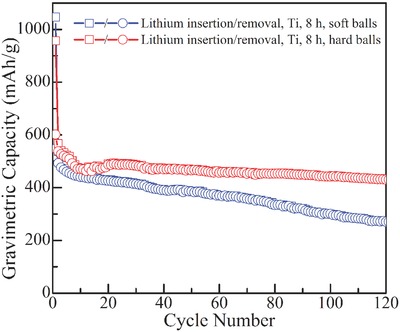
Cycling curves (cycled under C/10) of nanosized Sn‐Fe‐C anode materials synthesized with titanium as reducing agents and different grinding media indicated by each curve (total ball mill time is 8 h).

The electrochemical cycling curves of the 8 h samples comparing the use of soft and hard balls are shown in **Figure** **3**. It is clear that the material synthesized by hard ball grinding media delivers reversible gravimetric capacity of ≈500 mAh g^−1^ and excellent capacity retention over 120 cycles, much better than the soft balls grinding media. Thus, hard iron balls are chosen as the grinding media to prepare the materials for the following studies.

### 2.3 Graphite Content

Graphite is added during the synthesis since carbon can serve multiple functions during the ball milling process, such as promoting the formation of amorphous materials, modifying the materials microstructure, as well as increasing the specific capacity.[[qv: 4a,22]] Our experiments have shown that if no graphite is added, the product is still a mixture of tin and Sn_2_Fe (Figure S2a, Supporting Information). However, the tin in the product melted due to the high temperature caused by the high energy ball milling process and then stuck on the wall of the ball milling vial (Figure S2b, Supporting Information). It is very difficult to remove the product from the ball milling vial and thus this approach is not feasible to make electrodes.

Therefore, this mechanochemical synthesis should not be carried out without the addition of carbon. The well‐defined Bragg peaks corresponding to graphite cannot be clearly identified after grinding, which is probably due to the low crystallinity of graphite after being subjected to high energy ball‐milling, consistent with an earlier report.^23^

**Figure 4:**
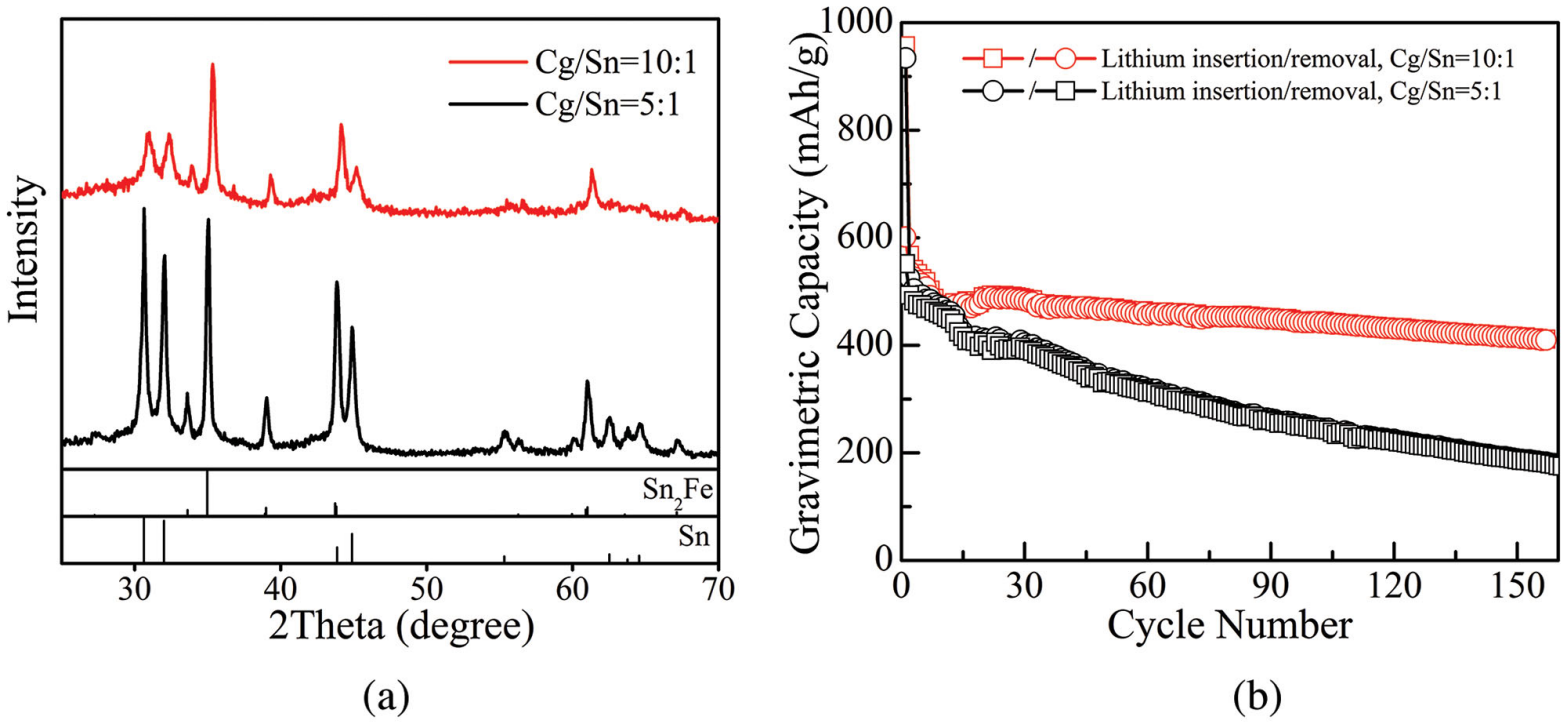
a) XRD patterns and b) cycling performances (cycled under C/10) of nanosized Sn‐Fe‐C anode materials synthesized by different graphite (Cg) to tin ratio.

The impact of varying the graphite/Sn molar ratio used in synthesizing the Sn‐Fe‐C composite was determined. Reducing the graphite content from 10:1 to 5:1 increases the crystallinity of the products formed, as shown in **Figure** **4**a. This suggests that reducing the carbon content enhances the sintering of the tin particles, which would be expected to lead to a poorer electrochemical behavior. Indeed, as shown in Figure 4b, the capacity retention of the 5:1 composite is much worse than the 10:1 material. Thus reducing the graphite content is not recommended.

As noted above when the graphite/tin ratio decreases from 10:1 to 5:1, the components of the final product do not change. However, their ratio does change. The composition of the final product from refinement results (Figure S3, Supporting Information) shows that the tin/Sn_2_Fe ratio increases with decreasing graphite/tin ratio, which explains the fast capacity degradation of the capacity. The carbon plays a key role in the composite, just as in the SONY SnCo anode,^13^ providing a coating for the active material protecting it from the electrolyte and providing a matrix in which the expansion and contraction of the active material is cushioned.

### 2.4 Different Carbon Type

In order to investigate the influence of the carbon type on the electrochemical performance of the final product, ethylene black was used to substitute for part or all of the graphite in the synthesis.

**Figure 5:**
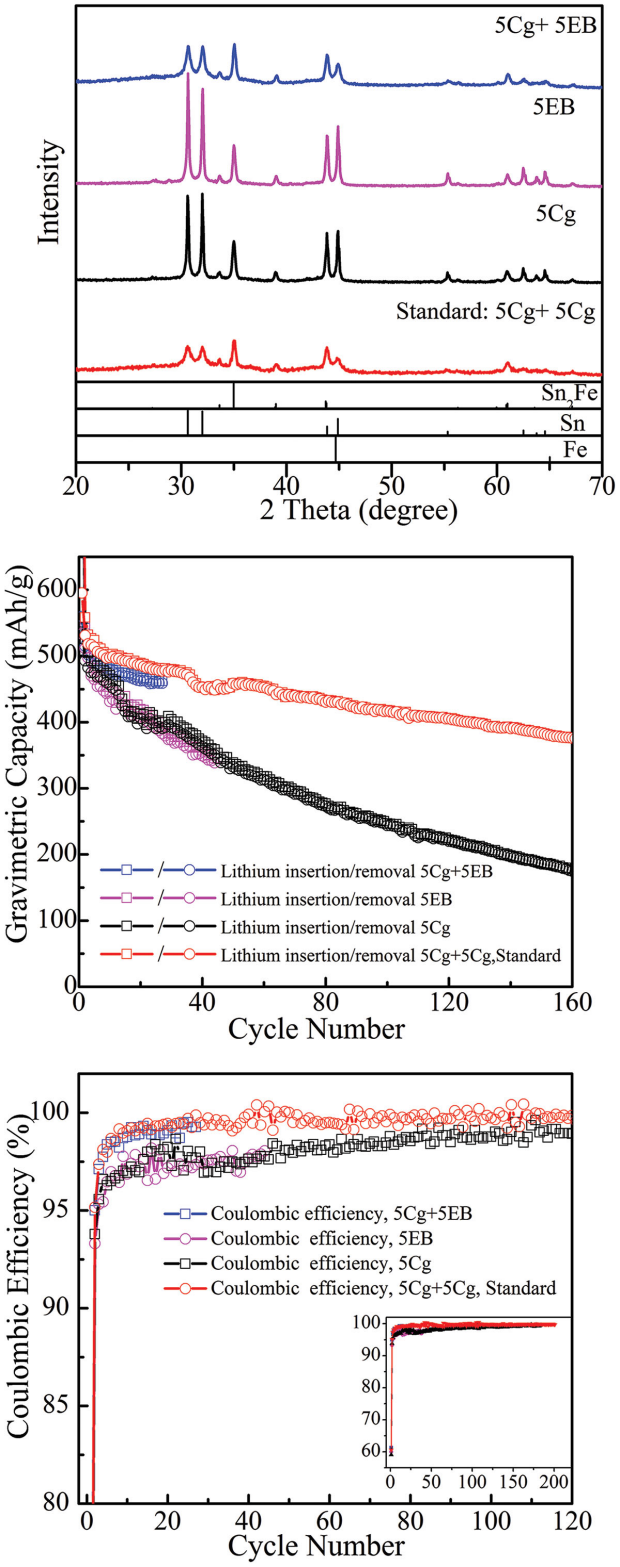
XRD patterns (upper), cycling performances (middle), and coulombic efficiency (lower) of nanosized Sn‐Fe‐C anode materials synthesized by different types of carbon (graphite is denoted as Cg while ethylene black denoted as EB). The inset shows the same coulombic efficiency plot as the lower figure including the first cycle.

The diffraction patterns show that all the products contain both crystalline tin and Sn_2_Fe, but less carbon leads to larger amounts and greater crystallinity of the elemental tin (**Figure** **5** (top)). Thus, whether graphitic carbon or carbon black was used had no discernible impact on the diffraction pattern; the two carbons acted in a similar manner. Similarly, the type of carbon does not impact the electrochemical behavior, which is also shown in Figure 5. Both the capacity retention and the cycling efficiency are the same for both 10:1 composites and these are much superior to the 5:1 composites. Thus, the quantity, but not the type, of carbon matters. Graphite was retained in the synthesis process because of the slightly higher capacity observed for it.

### 2.5 Ti Content

**Figure 6:**
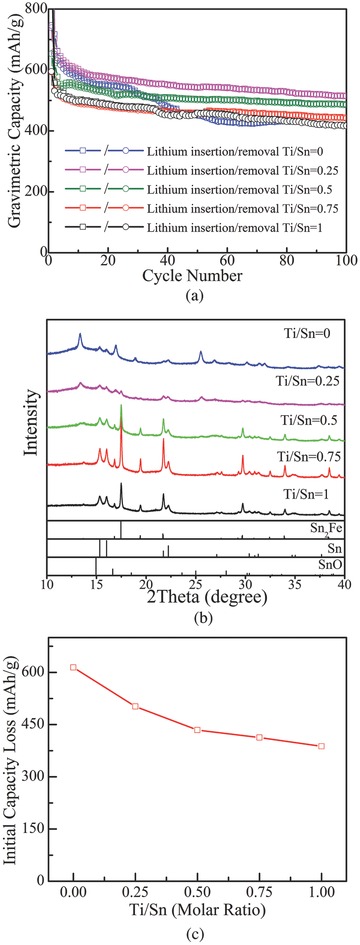
a) Electrochemical performance (cycling at C/10), b) synchrotron XRD patterns (wavelength = 0.779 Å), and c) first cycle capacity loss of nanosized Sn‐Fe‐C synthesized by mechanochemical method with different Ti/Sn ratios.

The Ti/Sn molar ratio used in the synthesis was varied to determine the optimum condition. As shown in **Figure** **6**a, that after a few cycles, a low but not zero amount of Ti leads to the highest capacity and capacity retention. The capacities here include the weight of the titanium, and so it is no surprise that the highest Ti contents lead to the overall lowest capacities. However, the Ti free composition, although having the highest initial capacity fades more quickly than the others. Figure 6b suggests that the Ti content also plays a role in the crystallinity of the material; the lowest Ti content materials are the least crystalline.

A significant issue with many of the substitutes for the carbon anode is the first cycle capacity loss, i.e., the difference in capacity between the first and subsequent cycles. For these tin‐based materials this loss decreases continuously from 610 to 390 mAh g^−1^ as the Ti/Sn ratio increases (Figure 6c). Just a small amount of Ti seems to be effective, increasing it above a ratio of 0.5 appears to have diminishing returns. It is important to point out that this is not in this case strictly a loss in capacity, but an excess in capacity during the first cycle. If Sn, Sn_2_Fe, and C are considered as active materials, the theoretical capacity of the composite synthesized under standard conditions is 509 mAh g^−1^.

The capacity after the first cycle is greater than that expected from the reactions of Li with Sn and carbon to form Li_4.4_Sn and LiC_6_. The reaction of Li with some of the carbon to form the highly saturated lithium‐graphite compound LiC_2_ must be invoked.[[qv: 22b]] However, the first lithium insertion capacity still exceeds the theoretical maximum capacity even considering all the Sn, Sn_2_Fe, and C as active materials. This suggests that side reactions take place on the surface of the particles to form a solid electrolyte interphase (SEI) layer, which is prevalent for tin‐based anodes.^24^ Moreover, contributions from the surface oxidation could not be ruled out; especially the titanium oxides in this composite since the lithium titanium oxide formed in the first discharge is not reversible at the charging potentials used.^25^ Defective carbon resulted from high energy milling is believed to be another major contributor for the first cycle excess capacity, which is supported by Matsumura's results that lithium can be not only located between graphitic layers but also located at the edge of graphitic layers and on the surface of crystallite in disordered carbon formed from ball mill process.^26^

Due to the complex nature of the composite prepared by high energy ball milling (a mixture of pure Sn, Sn_2_Fe, defective graphite, titanium oxides, etc.), the precise reasons causing the first cycle excess capacity as well as the methods to mitigate it are still under study. However, the formation of an SEI layer on the reactive products formed, and reaction with any residual SnO to form Li_2_O and lithiation of Ti oxides are key contributors, as the latter reactions are not reversible at the anode potentials used.

From an overall capacity retention point of view, an optimum Ti/Sn ratio appears to be between 0.25 and 0.5, and we chose 0.25 as our optimum Ti/Sn ratio since it exhibits a higher reversible capacity than 0.5.

### 2.6 Optimum Synthesis Method

**Figure 7:**
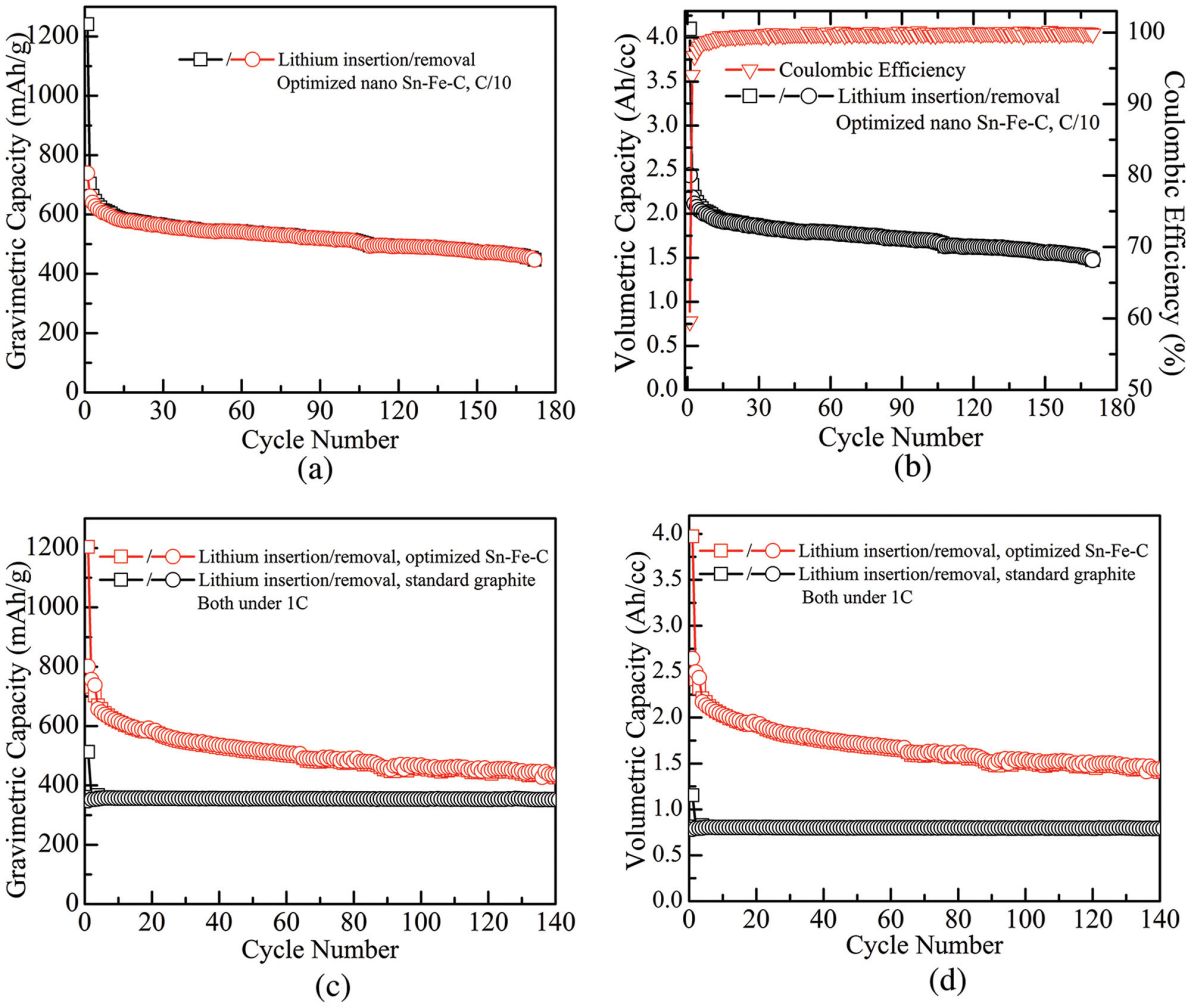
Electrochemical performance of the optimized Sn‐Fe‐C composite cycled under C/10 is shown by a) gravimetric capacity and b) volumetric capacity with coulombic efficiency. c) Gravimetric capacity and d) calculated volumetric capacity comparison of the optimized Sn‐Fe‐C composite with standard graphite cycled under 1 C rate, with a current density of 3.12 mA cm^−2^ for Sn‐Fe‐C composite and 1.75 mA cm^−2^ for standard graphite (1 C corresponding to a current density of 600 mA g^−1^ for Sn‐Fe‐C, 372 mA g^−1^ for standard graphite). The efficiency is well over 99% after the first few cycles in (d).

Based on above studies, the optimum mechanochemical synthetic conditions are mixing SnO, Ti, and graphite in an initial ratio of 1:0.25:10 and ball‐milling for 8 h using hard balls. The measured tap density of this optimized material was 1.8 g cc^−1^, compared to less than 1.0 g cc^−1^ for the graphite used; its measured surface area was 9.7 m^2^ g^−1^. When cycled at a C/10 rate, such an optimized nano Sn‐Fe‐C composite can deliver a capacity of 740 mAh g^−1^ (≈2.4 Ah cc^−1^) for the first charging process along with excellent retention over 170 cycles, while 60% of its original capacity still remains after 170 cycles (**Figure** **7**a). The calculated volumetric capacity exceeds 1.6 Ah cc^−1^ for 140 cycles, which is around double the capacity of carbon and the cycling efficiency exceeds 99% for most of the cycles (Figure 7b). The volumetric capacity was calculated based on the amounts and the densities of the reaction products, not of the initial reactants. At a C rate on both discharge and charge the capacity retention of optimized Sn‐Fe‐C still exceeds that of a carbon anode when cycled under the same conditions for more than 140 cycles (Figure 7c,d). Beyond 150 cycles the lithium electrode failed, but on replacement cycling could be continued beyond 200 cycles (see Figure S4, Supporting Information).

## 3 Conclusion

Nanosized Sn‐Fe‐C composites were successfully synthesized through a mechanochemical route, and the impacts of several key factors in this synthesis were evaluated. Materials formed using hard grinding balls have better electrochemistry than those using soft balls. The optimum carbon and titanium content as well as the total grinding time in the synthesis were determined to be: initial ratio of SnO, Ti, and graphite of 1:0.25:10, and high energy ball milling of 8 h. These conditions deliver overall electrochemical performance of higher than 500 mAh g^−1^ for at least 100 cycles at C/10. The calculated volumetric capacity remains higher than 1.6 Ah cc^−1^ over 140 cycles, which is double that of graphitic carbon. This Sn‐Fe‐C composite exhibits excellent rate capability as well, delivering volumetric capacity higher than 1.6 Ah cc^−1^ over 140 cycles when cycled under 1 C rate, which shows all the capabilities to be a promising candidate replacing carbon anode in the future. However, the initial excess capacity must be understood and overcome.

## 4 Experimental Section

Tin‐iron based anode composites were synthesized based on our earlier approach.^19^ In this synthesis, SnO (powder, 97%, Sigma‐Aldrich), Ti (powder, −100 mesh, Sigma‐Aldrich), and graphite (power, <20 μm, Sigma‐Aldrich) with the molar ratio of 1:1:5 were put into a stainless steel vial with six 1/2 in. diameter and ten 1/4 in. diameter stainless steel balls, and then the vial was subjected to high energy ball milling (SPEX‐8000) for 2 h. After which, an equal amount of graphite as before was added to the vial and then the vial was ball milled for another 6 h. The terms hard and soft are used to represent the SPEX hardened steel balls and Across International steel balls, respectively; the soft balls can be deformed under 5 tons pressure whereas the hard ones did not show a visible deformation under this pressure.^19^ To avoid oxidation during the milling process, the chemicals and vial were handled under purified helium atmosphere before milling, in an MBRAUN glove box with O_2_ and H_2_O contents at ppm levels.

The XRD patterns were collected on a Scintag XDS2000 powder diffractometer (*λ* = 1.54178 Å). For synchrotron XRD in Figure 6b and Figure S3 (Supporting Information), the powder samples from as‐prepared electrodes were scraped off, filled in separate capillaries, and characterized at the beamline 14A (wavelength 0.779 Å) at National Synchrotron Light Source (NSLS), Brookhaven National Laboratory (BNL). Data refinement and analysis was done with General Structure Analysis System (GSAS).^27^ The morphology of the sample was studied by a Zeiss Supra 55 VP field emission SEM operating at 5 kV.

TEM microstructural images of the Sn‐Fe‐C nanoparticles were acquired at Brookhaven National Laboratory, using JEM‐2100F (JEOL) operated at an acceleration voltage of 200 kV. For the TEM samples, the Sn‐Fe‐C nanoparticles (powder) were mixed in the DMC solution in a small vial, and sonicated for 2–3 min. The well‐dispersed nanoparticle solution was then dispersed onto a lacey carbon TEM grid for imaging.

XPS of the samples was performed using a laboratory based monochromated Al Kα source with a hemispherical analyzer at the Analytical and Diagnostics Laboratory (ADL) at Binghamton University. The Cr 2p region was measured with a pass energy of 23.5 eV, corresponding to an instrumental resolution of 0.51 eV determined from both the Au 4f7/2 core level and Fermi edge of the Au foil.

For the electrode preparation, 80 wt% active material, 10 wt% carbon black, and 10 wt% polyvinylidene fluoride (PVDF) binder were mixed with appropriate amount of *N*‐methyl‐2‐pyrrolidone (NMP) solvent to form slurry. The obtained slurry was spread onto copper foil by a doctor‐blade and then dried in vacuum oven at 80 °C overnight. The electrodes (with active material mass around 5 mg) were assembled into 2325‐type coin cells in an He‐filled glove box with lithium foil (Aldrich, thickness 0.38 mm) as the counter electrodes and Celgard 2400 separator (Hoechst Celaese). The electrolyte was 1 m lithium hexafluorophosphate (LiPF_6_) dissolved in ethylene carbonate (EC) and dimethyl carbonate (DMC) with volume ratio of 1:1 and 10 % fluoroethylene carbonate (FEC) as additive. The electrode samples were loaded with 4−6 mg of active material.

The electrochemical performance was tested using a VMP multichannel potentiostat (Biologic). The galvanostatic cycling tests were performed at various C‐rate (1 C corresponding to a current density value of 600 mA g^−1^) within 0.01–1.50 V voltage limit.

## Supporting information

As a service to our authors and readers, this journal provides supporting information supplied by the authors. Such materials are peer reviewed and may be re‐organized for online delivery, but are not copy‐edited or typeset. Technical support issues arising from supporting information (other than missing files) should be addressed to the authors.

Supplementary
