# Supplementary material for: The Anode Challenge for Lithium‐Ion Batteries: A Mechanochemically Synthesized Sn–Fe–C Composite Anode Surpasses Graphitic Carbon
Source: Adv Sci (Weinh). 2016 Feb 4;3(4):1500229. doi: 10.1002/advs.201500229 (PMC5067663; doi:10.1002/advs.201500229)
Supplement: Supplementary file 1 — Supplementary [file ADVS-3-0a-s001.pdf]

Copyright WILEY-VCH Verlag GmbH & Co. KGaA, 69469 Weinheim, Germany, 2016.

ADVANCED SCIENCE
Open Access

# Supporting Information

for *Adv. Sci.*, DOI: 10.1002/advs.201500229

# The Anode Challenge for Lithium-Ion Batteries: A Mechanochemically Synthesized Sn–Fe–C Composite Anode Surpasses Graphitic Carbon

*Zhixin Dong, Ruibo Zhang, Dongsheng Ji, Natasha A. Chernova, Khim Karki, Shawn Sallis, Louis Piper, and M. Stanley Whittingham**

Copyright WILEY-VCH Verlag GmbH & Co. KGaA, 69469 Weinheim, Germany, 2013.

# Supporting Information

## The anode challenge for lithium-ion batteries: a mechanochemically synthesized Sn-Fe-C composite anode surpasses graphitic carbon

*Zhixin Dong, Ruibo Zhang, Dongsheng Ji, Natasha A. Chernova, Khim Karki, Shawn Sallis, Louis Piper and M. Stanley Whittingham**

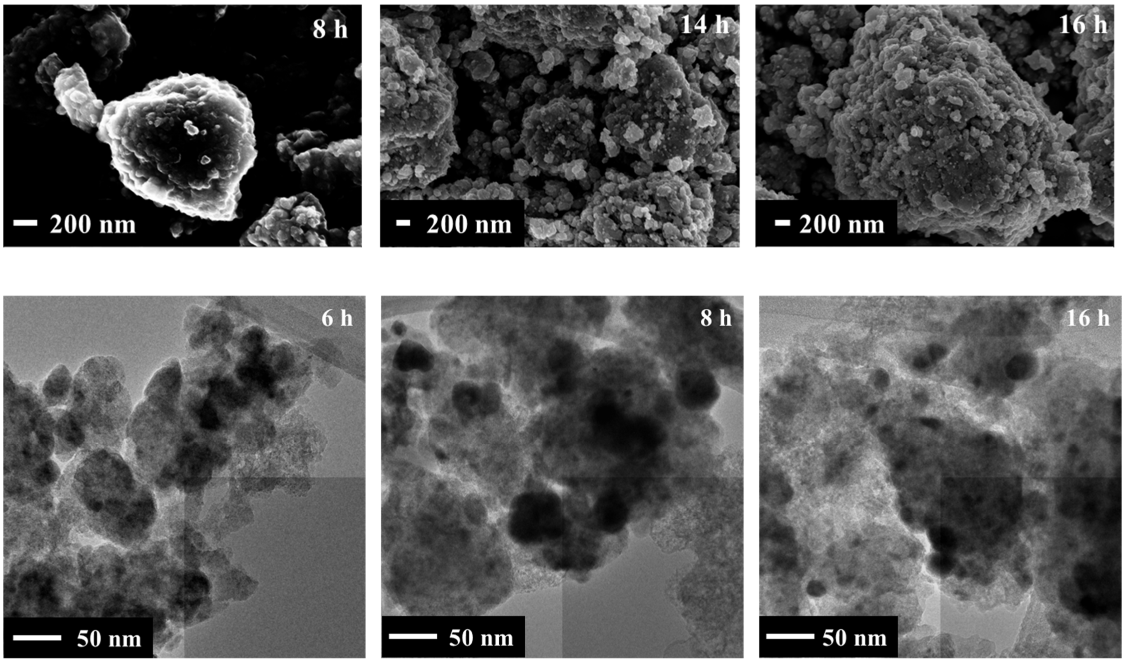


**Figure S1.** SEM (upper) and TEM (bottom) images of nanosized Sn-Fe-C anode materials synthesized by different total grinding time.

The sample is composed of nanosized primary particles of less than 100 nm in diameter and aggregates to form big micro-sized particles. Longer grinding time leads to larger aggregates of smaller nanoparticles.

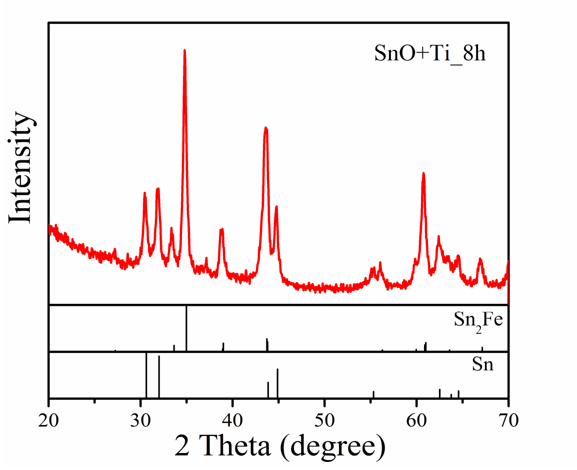


(a)

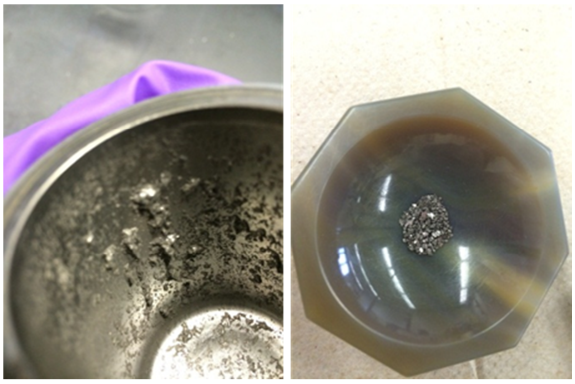

(b)

**Figure S2.** (a) XRD pattern and (b) photos of the product of SnO and Ti high energy ball milled by 8h, without graphite added. Without the addition of graphite, the product sticks on the ball milling vial.

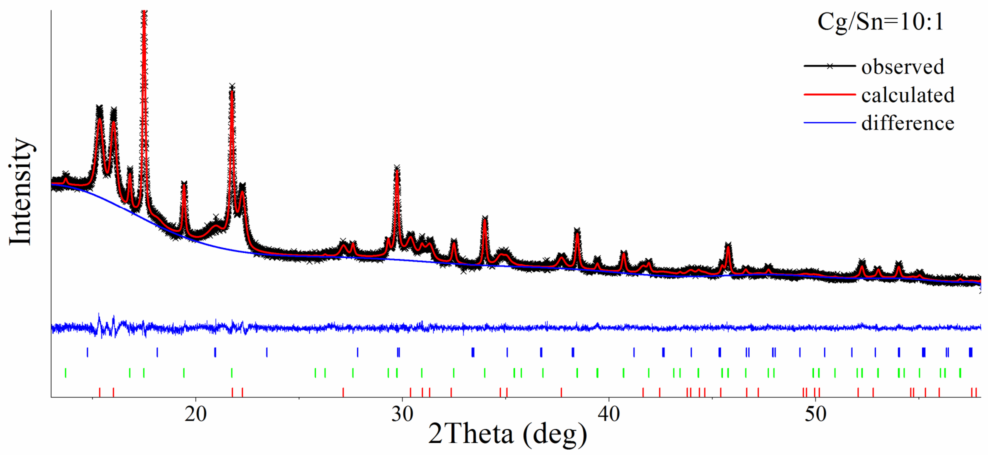


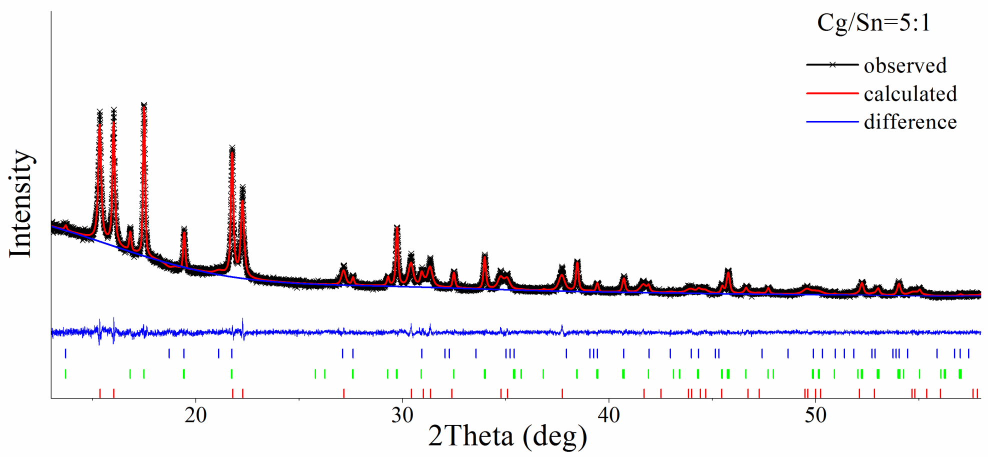


| | **Percentage (molar) when Cg/Sn = 10:1** | **Percentage (molar) when Cg/Sn = 5:1** |
|---|---|---|
| **Sn** | 47% | 57% |
| **$Sn_2Fe$** | 28% | 18% |
| **$TiO_2$** | 25% | 25% |

**Figure S3.** Rietveld refinement of synchrotron XRD data (wavelength = 0.779 Å) of Sn-Fe-C anode material prepared by ball-milling with graphite to tin ratio of 10:1 (upper), 5:1 (middle) and corresponding tables of compositions (bottom). Red bar: Sn; Green: $Sn_2Fe$; Blue: $TiO_2$.

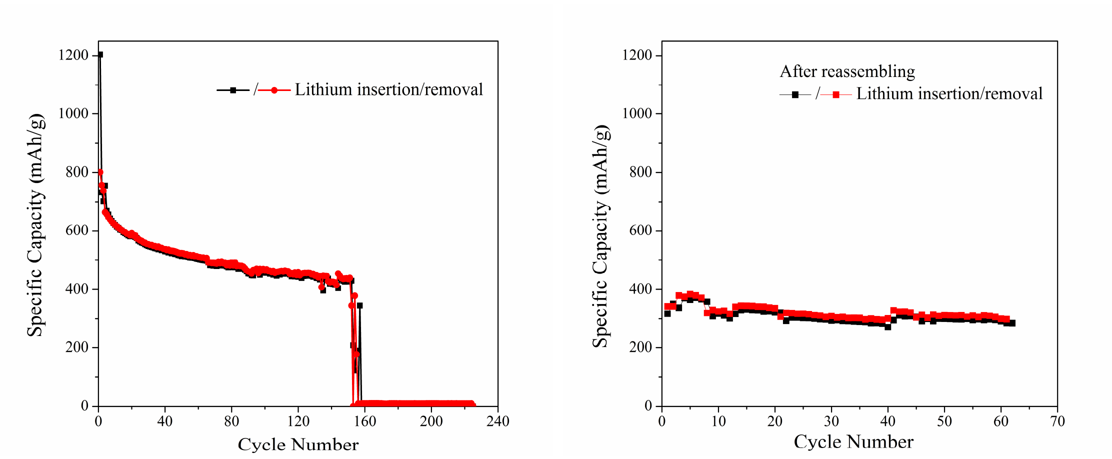


Figure S4. The cycling curves of optimized Sn-Fe-C composite (left) before and (right) after replacing lithium reference electrode in the coin cell.
